# Enteroscopic sclerotherapy in blue rubber bleb nevus syndrome

**DOI:** 10.12669/pjms.311.5858

**Published:** 2015

**Authors:** Shoubin Ning, Yafei Zhang, Zhanfei Zu, Xuyan Mao, Gaoping Mao

**Affiliations:** 1 Shoubin Ning, Department of Gastroenterology, General Hospital of Air Force, PLA, Beijing, China.; 2 Yafei Zhang, Department of Gastroenterology, General Hospital of Air Force, PLA, Beijing, China.; 3 Zhanfei Zu, Department of Gastroenterology, General Hospital of Air Force, PLA, Beijing, China.; 4 Xuyan Mao, Department of Gastroenterology, General Hospital of Air Force, PLA, Beijing, China.; 5 Gaoping Mao, Department of Gastroenterology, General Hospital of Air Force, PLA, Beijing, China.

**Keywords:** Blue rubber bleb nevus syndrome, Vascular malformation, Enteroscope, Sclerotherapy

## Abstract

Blue rubber bleb nevus syndrome (BRBNS) is a rare syndrome characterized by multiple vascular malformations of varying size and appearance that present predominantly on the skin and within the gastrointestinal tract and, less often, in other internal organs. Gastrointestinal lesions of BRBNS can cause acute or chronic bleeding, and the treatment is challenging. In this case, we reported a successful treatment of vascular malformations in all segments of gastrointestinal tract, including the small intestine, by endoscopic sclerotherapy, in a 10-year-old boy with BRBNS.

## INTRODUCTION

Blue rubber bleb nevus syndrome (BRBNS) is a rare systemic disorder with characteristic vascular malformations of the skin, gastrointestinal tract, and, less often, other internal organ.^1^ Over 250 cases of BRBNS have been reported in the English literature since Gascoyen’s first presentation in 1860.^2^ The skin lesions in BRBNS are often first noticed at birth or in the neonatal period, but rarely cause serious clinical problems which must be managed. Gastrointestinal hemangiomas of BRBNS are the most clinically relevant malformations, which can cause acute or chronic bleeding, with consequent iron deficiency, anemia, and even death.

The gastrointestinal hemangiomas of BRBNS are usually distributed throughout the gastrointestinal tract and mostly in the small intestine and distal colon.^3^ Endoscopic therapies for these gastrointestinal lesions of BRBNS have been attempted such as laser photocoagulation, sclerosis and band ligation, etc.^4^ In this manuscript, we report a successful treatment of vascular malformations in all segments of gastrointestinal tract, including the small intestine, by endoscopic sclerotherapy, in a 10-year-old boy with BRBNS.

## CASE REPORT

**Fig.1:**
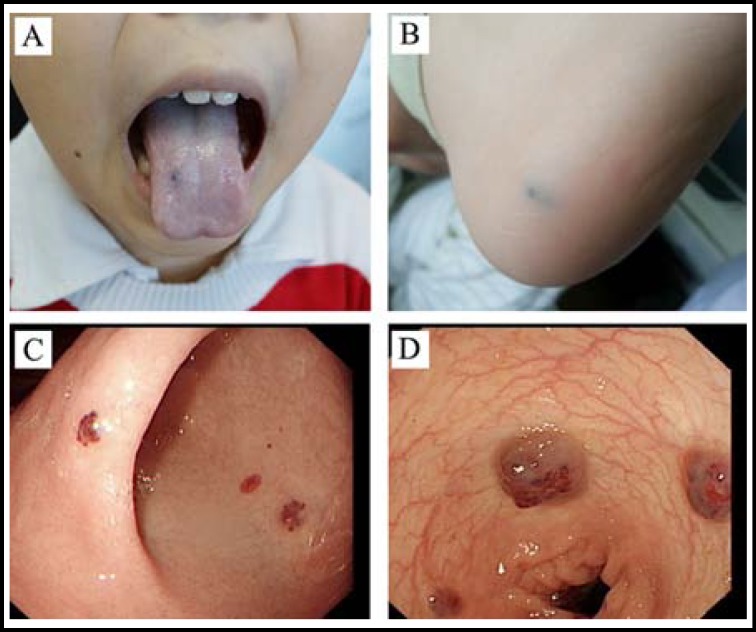
(A) The lesion in tongue; (B) The lesion in the sole of left foot; (C) The lesions in stomach; (D) The lesions in colon.

The patient was a 10-year-old boy with no family history of BRBNS. At birth, no obvious abnormality was noted. From 4-year-old, the child was gradually found to have multiple blue nevus in the tongue and the skins on the sole of left foot, right forearm, neck and genitalia, etc (Fig. 1A and 1B). From 6-year-old, he began to present with recurrent melena, and sometimes dark red bloody stool, often resulting in severe anemia with hemoglobin levels that even dropped blow 35 g/L. Gastroscopic and colonoscopic examination in the local hospital revealed multiple hemangiomas in the esophagus, stomach and colon (Fig. 1C and 1D), and therefore in combination with the skin lesions, the child was diagnosed as BRBNS. In 2012, the child patient received therapeutic sclerotherapy several times for the gastrointestinal vascular malformations by using gastroscope and colonoscope, in which about total 20 hemangiomas were treated. However, after the treatment, he still presented with recurrent melena and bloody stool, and consequent anemia.

From the beginning of 2013, the child began to receive therapy in our hospital. Examinations by enteroscopy revealed over 60 hemangiomas ranging in size from 3 mm to 30 mm throughout the digestive tract, among which the distribution was highest in duodenum, upper jejunum and distal ileum. Under general anesthesia using propofol, the child patient was treated with enteroscopic sclerotherapy for the hemangiomas in the gastrointestinal tract, especially in the small intestine. By lauromacrogol (also known as polidocanol, 100 mg/10 ml, Shaanxi Tianyu Pharmaceutical Co., Ltd., China) injection, total 42 hemangiomas in the small intestine and other 20 hemangiomas in the stomach and colon were treated (1~3 ml per lesion). Most of the hemangiomas disappeared completely after one injection of lauromacrogol. For the residual lesions observed in the next enteroscopy, an additional injection could be given.

**Fig.2:**
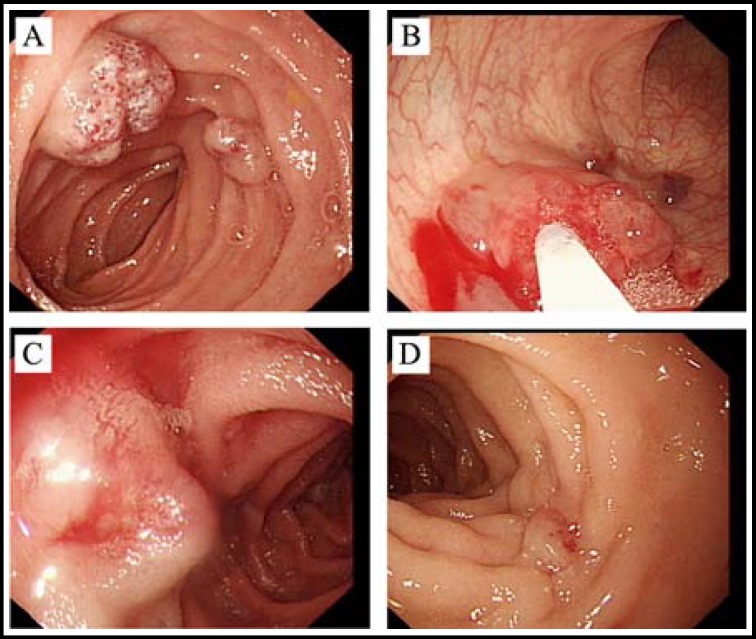
(A) The lesions in small intestine; (B) Lauromacrogol injection by enteroscopy; (C) The lesion after lauromacrogol injection was white and swollen; (D) Scar formation observed about 6 months late

In this patient, over 2/3 of the gastrointestinal lesions were located in the small intestine, and for which, the treatment of the small intestinal lesions was particularly appealing. A total of 8 sessions of enteroscopy (5 times by single-balloon enteroscope in the first round of therapy and 3 times by double-balloon enteroscope in the second round of therapy) were performed on this patient. In the therapeutic process, minor exudative hemorrhage could be observed in the site of injection and after infusion of norepinephrine, the hemorrhage usually stopped immediately. No severe therapy-related adverse events such as postoperative reactionary hemorrhage and intestinal perforation was noticed. Fig.2 shows the general process of enteroscopic sclerotherapy in this patient.

After two rounds of therapy in our hospital, melena and bloody stool have been absent in this young patient for almost 6 months and at present the hemoglobin has recovered to normal level.

## DISCUSSION

The cause of BRBNS is still not clear. Some evidence suggests that it may be due to the inheritance of some defective genes, but in most cases the disease appears to occur randomly.^5^ BRBNS can occur at any age and does not have a sex predilection.^6^ In children with BRBNS, because of the lack of specific signs and symptoms, their families often ignore the existence of the disease. Only after development of severe melena and anemia, these children will be drawn to seek help from hospital.

The diagnosis of BRBNS was established by clinical history, physical appearance of cutaneous lesions, imaging studies, and the pathognomonic endoscopic appearance of the gastrointestinal lesions. At present, no curative therapy is available. Pharmacologic agents including corticosteroids and interferon-α have been used in an attempt to control the gastrointestinal bleeding in BRBNS, and however, it is unlikely to be of any benefit. Conservative treatment such as iron replacement and transfusion is clinically most feasible, but it may be a life-long therapy for these patients, which is expensive and decreases the quality of life. There is no need for surgical intervention in most cases because the lesions are often distributed throughout the digestive tract. Only in a life-threatening situation, surgery may be the optional treatment. Endoscopic interventions, such as argon plasma coagulation, laser photocoagulation, electrocauterization, band ligation and sclerosis, have been considered as playing a critical role in the treatment of BRBNS. However, some endoscopists considered that the wall of small intestine is relatively thin and therefore suggested the endoscopic interventions such as argon plasma coagulation for lesions in small intestine should be prudent.^7^ In this case, we chose to perform sclerotherapy with injection of lauromacrogol for the small intestinal lesions by enteroscopy. Total 42 hemangiomas located in the small intestine were treated, and no one therapy-related adverse event happened, indicating the safety of sclerotherapy as similar as that for gastric or colonic lesions.

It should be noted that the child patient received two rounds of therapy in our hospital. Between January and March of 2013, the child received total 5 times sclerotherapy for the small intestinal hemangiomas by single-balloon enteroscope including 3 times peroral (maximal 100 cm into jejunum) and 2 times peranal (maximal 150 cm into ileum). However, after about half a year, the child was admitted to our hospital again for bloody stool. Between October and December of 2013, the child received another 3 times sclerotherapy for the small intestinal hemangiomas by double-balloon enteroscope including 2 times peroral (maximal 200 cm into jejunum) and 1 peranal (maximal 250 cm into ileum). This time, another 14 hemangiomas in the deep small intestine were treated. Unfortunately, complete exploration of the small intestine was still not accomplished. In addition, in this patient, newly formed hemangiomas in the gastrointestinal tract could be observed under endoscopy, even just after a few months. Therefore, the child patient is still under our clinical observation, and further treatment may be needed.
